# Late-gadolinium enhancement is common in older pediatric heart transplant recipients and is associated with lower ejection fraction

**DOI:** 10.1186/s12968-023-00971-8

**Published:** 2023-11-06

**Authors:** Andrew A. Lawson, Kae Watanabe, Lindsay Griffin, Christina Laternser, Michael Markl, Cynthia K. Rigsby, Melanie Sojka, Joshua D. Robinson, Nazia Husain

**Affiliations:** 1 Division of Cardiology, Department of Pediatrics, Ann & Robert H. Lurie Children’s Hospital of Chicago, Northwestern University Feinberg School of Medicine, Chicago, IL USA; 2 Department of Medical Imaging, Ann & Robert H. Lurie Children’s Hospital of Chicago, Chicago, IL USA; 3 Department of Radiology, Northwestern University Feinberg School of Medicine, Chicago, IL USA; 4 Center for Cardiovascular Innovation, Ann & Robert H. Lurie Children’s Hospital of Chicago, Chicago, IL USA; 5 Department of Pediatrics, Baylor College of Medicine, Houston, TX USA

**Keywords:** Pediatric heart transplant, Cardiac MRI, Late gadolinium enhancement

## Abstract

**Background:**

Chronic graft failure and cumulative rejection history in pediatric heart transplant recipients (PHTR) are associated with myocardial fibrosis on endomyocardial biopsy (EMB). Cardiovascular magnetic resonance imaging (CMR) is a validated, non-invasive method to detect myocardial fibrosis via the presence of late gadolinium enhancement (LGE). In adult heart transplant recipients, LGE is associated with increased risk of future adverse clinical events including hospitalization and death. We describe the prevalence, pattern, and extent of LGE on CMR in a cohort of PHTR and its associations with recipient and graft characteristics.

**Methods:**

This was a retrospective study of consecutive PHTR who underwent CMR over a 6-year period at a single center. Two independent reviewers assessed the presence and distribution of left ventricular (LV) LGE using the American Heart Association (AHA) 17-segment model. LGE quantification was performed on studies with visible fibrosis (LGE+). Patient demographics, clinical history, and CMR-derived volumetry and ejection fractions were obtained.

**Results:**

Eighty-one CMR studies were performed on 59 unique PHTR. Mean age at CMR was 14.8 ± 6.2 years; mean time since transplant was 7.3 ± 5.0 years. The CMR indication was routine surveillance (without a clinical concern based on laboratory parameters, echocardiography, or cardiac catheterization) in 63% (51/81) of studies. LGE was present in 36% (29/81) of PHTR. In these LGE + studies, patterns included inferoseptal in 76% of LGE + studies (22/29), lateral wall in 41% (12/29), and diffuse, involving > 4 AHA segments, in 21% (6/29). The mean LV LGE burden as a percentage of myocardial mass was 18.0 ± 9.0%. When reviewing only the initial CMR per PHTR (n = 59), LGE + patients were older (16.7 ± 2.9 vs. 12.8 ± 4.6 years, p = 0.001), with greater time since transplant (8.3 ± 5.4 vs. 5.7 ± 3.9 years, p = 0.041). These patients demonstrated higher LV end-systolic volume index (LVESVI) (34.7 ± 11.7 vs. 28.7 ± 6.1 ml/m^2^, p = 0.011) and decreased LV ejection fraction (LVEF) (56.2 ± 8.1 vs. 60.6 ± 5.3%, p = 0.015). There were no significant differences in history of moderate/severe rejection (p = 0.196) or cardiac allograft vasculopathy (CAV) (p = 0.709).

**Conclusions:**

LV LGE was present in approximately one third of PHTR, more commonly in older patients with longer time since transplantation. Grafts with LGE have lower LVEF. CMR-derived LGE may aid in surveillance of chronic graft failure in PHTR.

## Background

The overall median survival for pediatric heart transplant recipients (PHTR) has improved over time and is now reaching 20 years [1]. The expected survival for an individual patient is multifactorial, dependent on both donor-recipient characteristics and acquired morbidity post-transplant [1, 2]. Graft failure, cardiac allograft vasculopathy (CAV), and acute rejection are the leading causes of death in PHTR greater than three years from the time of transplant [1]. As such, surveillance for the development of these disease processes is a critical element of post-transplant care. Surveillance methods in widespread clinical practice include cardiac catheterization (for hemodynamic assessment, coronary angiography, and endomyocardial biopsy), non-invasive molecular monitoring [3], and non-invasive cardiac imaging. Echocardiography remains the primary non-invasive imaging modality for PHTR, providing reproducible functional assessment and strain analysis [4]. Cardiovascular magnetic resonance imaging (CMR) confers the added benefit of non-invasive tissue characterization. Because CMR use in PHTR remains in an early stage, the literature is sparse. Understanding the significance of CMR-derived tissue parameters for PHTR, specifically, remains challenging.

Assessment for late-gadolinium enhancement (LGE) on CMR is a validated method to detect focal myocardial fibrosis [5], which has been associated with both chronic graft failure and cumulative rejection history on endomyocardial biopsies of PHTR [6]. In a gross clinical pathology study of 14 explanted heart grafts from PHTR, all grafts had significant epicardial fibrosis as well as some degree of subendocardial and myocardial fibrosis [7]. Although EMB allows for the direct, histologic evaluation for these myocardial changes, the procedure is invasive. Additionally, biopsies are typically limited to the right ventricular (RV) aspect of the interventricular septum. Assessment for LGE on CMR provides a means of non-invasive evaluation of fibrosis throughout the myocardium.

Among adult heart transplant recipients, the presence and extent of LGE are associated with increased risk of future adverse clinical events including hospitalization and death [8–11]. In these cohorts, LGE was common, present in 18–54% of patients [8–11]. LGE quantification methods were used to further describe the burden of LGE, as a percentage of left ventricular (LV) mass. The adult literature suggests a role for routine CMR with LGE assessment in the clinical surveillance of heart transplant recipients [8–10]. In smaller studies of CMR in PHTR to date the reported prevalence of LGE varies significantly from 0 to 67% [12–16] in cohorts with variable inclusion criteria, and the prognostic value of LGE for subsequent clinical outcomes has not been evaluated. In this study, we aimed to describe the prevalence, pattern, and extent of myocardial LGE in a cohort of PHTR at a pediatric hospital. Further, we hypothesized that presence of LGE may be associated with prior, adverse clinical events as well as changes in CMR-derived myocardial structural and functional parameters.

## Methods

### Study design and patient selection

This study is a cross-sectional, retrospective cohort study of PHTR who underwent comprehensive structure-function CMR with LGE at a single center from 2015 to 2021. The Institutional Review Board (IRB) approved this Health Insurance Portability and Accountability Act (HIPAA)-compliant study. PHTR were included with clinician ordered CMR and excluded if LGE sequences were incomplete or the quality was inadequate for analysis. Although all patients were followed at a pediatric center, age was not an exclusion criterion. We used all observations available for this study, which included multiple CMR studies for some patients.

### CMR protocol

CMR images were obtained according to a comprehensive, standardized protocol including structure and function analysis and delayed gadolinium-enhanced images [17]. All studies were performed on 1.5T scanners (Aera, Siemens Healthineers, Erlangen, Germany), with gadobutrol (Gadavist, Bayer HealthCare, Berlin, Germany) as the gadolinium-based contrast agent. A total gadobutrol dose of 0.15 mmol/kg was administered in up to three separate aliquots (if regadenoson stress perfusion imaging was performed), with LGE sequences performed 20–30 min from the time of initial contrast injection and at least 5 min after the last dose. For LGE sequences, the inversion time was selected using an inversion time scout scan to optimally null the normal myocardium. Segmented inversion-recovery sequences were obtained in three orientations: 4-chamber, 2-chamber, and short axis, from base to apex (TR = 2.8 ms, TE = 1.2 ms, slice thickness = 8 mm, flip angle 50°, in plane resolution = 1.4 × 1.4 mm^2^). In addition, 2D cine balanced steady-state free-precession (bSSFP) images were obtained in the 2-chamber, 3-chamber and 4-chamber, and short axis orientations (TR = 3.0 ms TE = 1.26–1.3 ms; flip angle = 90°, slice thickness = 6 mm, in plane resolution = 1.0 × 1.0 mm^2^.

### CMR post-processing-global cardiac volumes and function

CMR-derived structure and function analysis (including LV/RV end-diastolic and end-systolic volume indices (LV/RV EDVI and ESVI), LV mass index (LVMI), and LV/RV ejection fraction (EF) was performed at the time of completion of the CMR report using 2D bSSFP short-axis stack using dedicated commercially available software (Q Mass, Medis Suite 4.0.38.2, Medis Medical Imaging Systems, Leiden, The Netherlands). Ventricular volumes and LV mass were indexed to body surface area.

### CMR post-processing-LGE assessment

Two reviewers (LG and NH), determined the presence of LV LGE by visual identification of areas of relatively increased signal intensity on delayed-contrast enhanced images by consensus review. Orthogonal planes were used to confirm the presence of LGE. The reviewers assessed the distribution of LV LGE by the American Heart Association (AHA) 17-segment model and further classified the pattern as infarct-typical, with subendocardial involvement, or infarct-atypical, as described by Braggion-Santos et al. [18]. In addition, based on segmental distribution, three non-mutually exclusive descriptive patterns of LV LGE were observed: inferoseptal (posterior RV insertion point), lateral LV wall (involving AHA segments 5, 6, 11, 12, or 16), and diffuse (present in ≥ 4 LV AHA segments). Reviewers were blind to patient history.

For studies with LV LGE by qualitative assessment, two reviewers (AAL and NH) quantified the LGE burden by consensus review. Commercially available post-processing software (Q Mass, Medis Suite 4.0.38.2, Medis Medical Imaging Systems, Leiden, The Netherlands) was used. The reviewers selected 4–7 slices from base to apex in the short axis plane in which the myocardium was well visualized. Then, they manually traced LV epicardial and endocardial borders. The reviewers placed regions of interest in an area of healthy myocardium and an area of hyperenhanced tissue. Next, the LGE burden was quantified using the full-width half-maximum method. The LV borders were inspected again and adjusted, if necessary, to completely exclude the blood pool. LGE burden was recorded as a percentage of LV myocardium by mass ($\frac{LGE \, mass \left(g\right)}{Total \, LV \, mass\left(g\right)}\times 100\%$). Quantification was not performed if fewer than four short axis slices were analyzable. A single reviewer (NH), blinded to the prior results, performed a second round of LGE quantification in a subset of 5 LGE + patients to evaluate reproducibility.

### Clinical data collection

Demographic, clinical, and cardiac catheterization data were obtained by chart review. Clinical concern (e.g., active rejection, history of significant rejection, cardiac allograft vasculopathy, changes on echocardiogram, or changes in catheterization-derived hemodynamic parameters) was noted from clinical notes or CMR indication at the time the study was ordered. Patients were classified as having history of moderate to severe rejection if there was acute cellular rejection (ACR) ≥ 2R or antibody mediated rejection (AMR) ≥ 2 per International Society of Heart and Lung Transplantation criteria at any point since the most recent transplantation [19, 20]. The closest human leukocyte antigen—panel reactive antibody (HLA-PRA) prior to transplant was obtained, when available, and classified as > 10% or < 10%. The 10% threshold is a frequently used cut-point given the association between HLA-PRA > 10% and both AMR and decreased 1-year graft survival [21]. If hemodynamic catheterization and EMB were performed within 6 months of the CMR, the right atrial pressure, pulmonary capillary wedge pressure (PCWP), and EMB results were reported from the catheterization closest to the time of CMR. Patients were classified as having a history of CAV if there was any prior diagnosis of CAV from the time of transplantation through the annual catheterization with coronary angiography performed nearest to the time of CMR (before or up to 12 months after the CMR study). The interventional cardiologist performing the procedure graded angiograms for CAV according to International Society of Heart and Lung Transplantation criteria [22].

### Statistical analysis

We performed separate univariate analyses on the entire dataset of CMR studies as well as the set of initial CMR studies per PHTR, excluding follow-up studies. To test for significance, we used a t-test for continuous variables and Chi-squared test for categorical variables (Stata 17, College Station, TX, USA). We performed ordinary least squares regression analysis for presence of LGE with robust standard errors. We performed a Bland-Altman analysis to evaluate the agreement between LGE mass percentage values obtained by two blinded observers on 19% (5/27) of the LGE + cohort.

## Results

### Study population

During the study period, 81 CMR studies were performed on 59 unique PHTR. Reviewing the data for all CMR studies (n = 81), mean age at CMR was 14.8 ± 6.2 years with an average time since transplantation of 7.3 ± 5.0. Congenital heart disease was the most common indication for transplantation in this cohort 53% (43/81), followed by cardiomyopathy 35% (28/81), and re-transplantation 12% (10/81). A documented history of moderate/severe AMR or ACR prior to CMR was present in 29% (23/80) of all CMR studies, while a history of CAV was present in 11% (9/81). Specifically, there were 4 CMR studies of patients with CAV 1, two with CAV 2, and three with CAV 3. The indication for CMR was routine surveillance (without clinical concerns) in 63% of the studies (51/81). In the remaining 37% (30/81), a clinical concern was present. Clinical concerns, which were not mutually exclusive, included history of recent, significant rejection (n = 9) or CAV (n = 10), changes in catheterization-derived hemodynamic parameters (n = 4), changes in echocardiographic parameters (n = 3), LGE on prior CMR (n = 3), or unexplained cardiac symptoms (n = 2). The mean CMR-derived volumetric and functional parameters for all studies (n = 81) and initial studies (n = 59) are given in Table 1. Regadenoson-based stress perfusion was performed in 79/81 studies.

**Table 1:** CMR-derived mean volumes and tissue parameters for all CMR studies and for the subset of initial CMR studies only

	All CMR studies (n = 81)	Initial CMR studies only (n = 59)
LVEDVI, ml/m^2^ (SD)	75.0 (15.1)	74.6 (13.9)
LVESVI, ml/m^2^ (SD)	30.7 (9.2)	30.6 (8.7)
LV EF, % (SD)	58.7 (6.1)	59.1 (6.6)
LVMI, g (SD)	46.2 (10.4)	45.7 (9.0)
RVEDVI, ml/m^2^ (SD)	74.5 (15.8)	74.0 (15.3)
RVESVI, ml/m^2^ (SD)	33.4 (11.7)	32.8 (10.8)
RV EF, % (SD)	56.1 (7.1)	56.7 (8.8)
Qualitative LV LGE present, %	36 (29/81)	32 (19/59)
Qualitative RV LGE present, %	4 (3/81)	2 (1/59)

### LGE prevalence, pattern, and extent

**Fig. 1:**
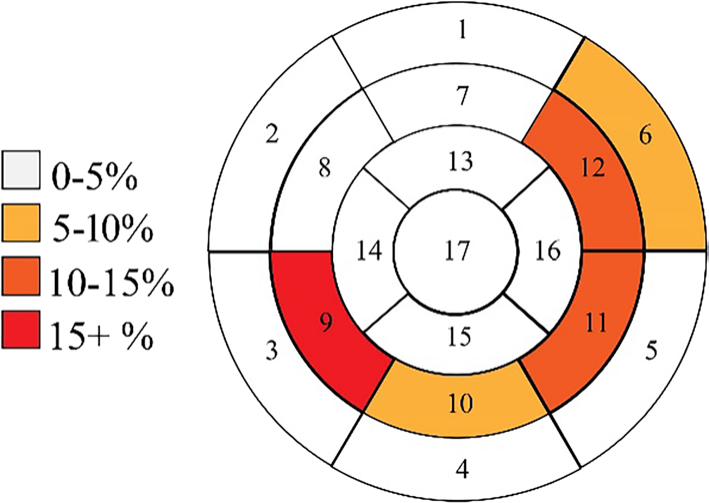
Frequency of LV LGE, by segment, using the AHA 17-segment model, on each patient’s initial CMR (n = 59)

**Fig. 2:**
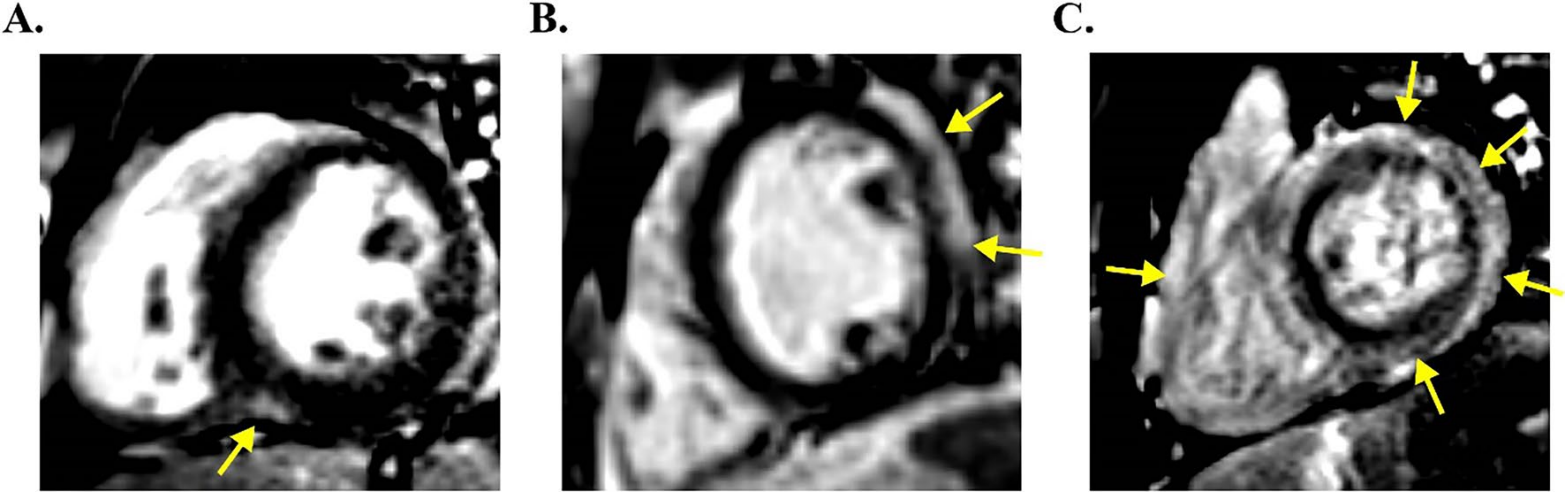
Observed infarct-atypical LGE patterns. CMR short axis images for 3 PHT with: **A** inferoseptal enhancement (RV insertion), **B** lateral-wall enhancement, and **C** diffuse LV enhancement (also with RV LGE). Areas of LGE are identified with arrows

LV LGE was present in 36% of all CMR (29/81) and 32% (19/59) of initial CMR per PHTR. Among LGE + scans, the mean number of AHA segments involved was 2.5 ± 2.7 (the segmental distribution is given in Fig. 1). The observed pattern was nearly exclusively infarct-atypical, in 97% (28/29) of LV LGE + studies. A single CMR showed an infarct-typical, subendocardial LGE pattern. That patient had CAV 3 with stenosis of the left main coronary artery with LGE in a basal-lateral and inferolateral distribution. Inferoseptal enhancement was most frequent, noted in 76% of LGE + studies (22/29), followed by lateral wall in 41% (12/29), and diffuse in 21% (6/29) (Fig. 2). RV LGE was found in 4% of all studies (3/81) and 2% of initial studies (1/59); each of these studies also had diffuse LV LGE.

Of the 29 CMR studies with qualitative LGE, two studies were disqualified from LGE quantification due to significant motion artifact precluding quantitative analysis. A median of 5.0 slices (range 4–7) were used for analysis. The mean LV LGE mass percentage was 18.0 ± 9.0% (range 4.1–37.0%) in the LGE + studies. A subset of 5 LGE + patients had repeat LGE quantification performed by a single reviewer to evaluate reproducibility of quantification results. A Bland-Altman analysis revealed no significant mean bias between the two observers for LV LGE mass percentage (− 0.01 ± 7.2% LGE); however, the 95% limits of agreement were wide (− 14.6 to 14.6% LGE).

### Associations of late gadolinium enhancement

#### With clinical history and catheterization data

When reviewing the initial CMR per PHTR (n = 59), LGE + PHTR were found to be older (16.7 ± 2.9 vs. 12.8 ± 4.6 years, p = 0.001), with a longer time since transplant (8.3 ± 5.4 vs. 5.7 ± 3.9 years, p = 0.041) (Table 2). There was no statistically significant difference in donor age at time of transplant between LGE + and LGE−  PHTR (12.0 ± 7.3 vs. 9.0 ± 7.7 years, p = 0.222). LGE + PHTR were more likely to have a cardiomyopathy history as the indication for transplant versus congenital heart disease or re-transplantation. Cardiomyopathy history was present in 58% of LGE + patients, compared to 25% LGE− patients (p = 0.027). Conversely, the LGE- group had more frequent history of congenital heart disease (63%) and re-transplantation (13%).

A clinical concern at the time of ordering the CMR was associated with the presence of LGE (47% of LGE + vs. 20% of LGE− , p = 0.030). Notably, there were no significant differences in history of moderate or severe rejection, history of any CAV, or hypertension diagnosis between LGE + and LGE- groups. There were six initial studies of patients with CAV, of which 3 had LGE, each in an infarct-atypical pattern. There were no significant group differences in PCWP, right atrial pressure, or histologic rejection grade by cardiac catheterization with EMB.

By regression analysis (Table 3), older age was correlated with the presence of LGE (β = 0.044, p = 0.001) independent of time since transplantation. Age and time since transplant were not correlated with each other (r = 0.069) and thus both variables were included in the model. Both time since transplantation (p = 0.051) and history of CAV (p = 0.054) nearly reached statistical significance for association with the presence of LGE.

#### With CMR-derived volumetric and functional parameters

The presence of LV LGE was associated with increased LV end-systolic volume index (34.7 ± 11.7 vs. 28.7 ± 6.1 ml/m^2^, p = 0.011) and lower LV EF (56.2 ± 8.1 vs. 60.6 ± 5.3%, p = 0.015). There was no difference in RVEDVI, RVESVI, or RV EF based on the presence of ventricular (LV and/or RV) LGE.

**Table 2:** Clinical and demographic associations with the presence of LGE on initial CMR

	N^a^	All patients	LGE +	LGE –	p-value
Demographics and graft characteristics					
Age, y, mean ± SD	59	14.1 ± 4.5	16.7 ± 2.9	12.8 ± 4.6	**0.001**
Female sex, n (%)	59	33 (56%)	8 (42%)	25 (63%)	0.14
Ischemic time, min, mean ± SD	51	209 ± 59	198 ± 49	213 ± 62	0.446
Donor age at transplant, y, mean ± SD	53	9.9 ± 7.6	12.0 ± 7.3	9.0 ± 7.7	0.222
Time since transplantation, y, mean ± SD	59	6.5 ± 4.7	8.3 ± 5.4	5.7 ± 3.9	**0.041**
Indication for transplant					
Cardiomyopathy, n (%)	59	21 (36%)	11 (58%)	10 (25%)	**0.027**
Congenital heart disease, n (%)		33 (56%)	8 (42%)	25 (63%)	
Re-transplantation, n (%)		5 (8%)	0 (0%)	5 (13%)	
Transplant comorbidities					
History of moderate or severe AMR or ACR, n (%)	58	16 (28%)	7 (39%)	9 (23%)	0.196
PRA > 10% at transplant, n (%)	37	17 (46%)	2 (22%)	15 (54%)	0.101
Hypertension diagnosis, n (%)	59	17 (29%)	7 (37%)	10 (25%)	0.881
Clinical concern present, n (%)	59	17 (29%)	9 (47%)	8 (20%)	**0.030**
History of any CAV, n (%)	59	6 (10%)	3 (16%)	3 (8%)	0.325
Cath/biopsy data					
Right atrial pressure, mmHg, mean ± SD	47	6.0 ± 4.3	6.3 ± 5.3	5.8 ± 3.6	0.709
PCWP, mmHg, mean ± SD	48	12.0 ± 5.8	13.6 ± 8.3	11.2 ± 3.8	0.177
Any rejection at time of cath, n (%)	48	13 (27%)	5 (29%)	8 (25%)	0.788
Global cardiac volumes and function (indexed)					
LVEDVI, ml/m^2^, mean ± SD	59	75 ± 14	78 ± 15	73 ± 13	0.190
LVESVI, ml/m^2^, mean ± SD	59	31 ± 9	35 ± 12	29 ± 6	**0.011**
LV EF, %, mean ± SD	59	59 ± 7	56 ± 8	61 ± 5	**0.015**
LVMI, g, mean ± SD	55	46 ± 9	48 ± 11	45 ± 8	0.208
RVEDVI, ml/m^2^, mean ± SD	58	74 ± 15	73 ± 14	74 ± 16	0.754
RVESVI, ml/m^2^, mean ± SD	58	33 ± 11	33 ± 11	33 ± 11	0.924
RV EF, %, mean ± SD	58	57 ± 8	56 ± 8	57 ± 8	0.689

Bold values denote statistical significance

*ACR* acute cellular rejection, *AMR* antibody mediated rejection, *CAV* cardiac allograft vasculopathy, *LVEDVI* left ventricular end-diastolic volume index, *LVESVI* left ventricular end-systolic volume index, *LV EF* left ventricular ejection fraction, *PCWP* pulmonary capillary wedge pressure, *RVEDVI* right ventricular end-diastolic volume index, *RVESVI* right ventricular end-systolic volume index, *RV EF* right ventricular ejection fraction, *SD* standard deviation

^a^Some data were not available for patients undergoing initial CMR, n = 59. Variation in total “n” is based on available data

**Table 3:** Predictors of LV LGE by multiple linear regression

	β (95% CI)	p-value
Age (y)	0.044 (0.020 to 0.068)	**0.001**
Time since transplant (y)	0.028 (– 0.00 to 0.055)	0.051
Moderate or severe rejection	0.101 ( – 0.156–0.358)	0.434
History of CAV	0.269 (– 0.004 to 0.542)	0.054
Hypertension diagnosis	0.18 (– 0.080 to 0.439)	0.171
Constant	– 0.586 (– 0.897 to – 0.274)	**0.000**

Bold values denote statistical significance

## Discussion

In this single-center cohort of PHTR undergoing CMR, LV LGE was a common finding, present in 36% of all CMR studies and 32% of initial studies. RV LGE was observed rarely, in 4% of all studies in 2% of initial studies. LV LGE + PHTR were older with greater time since transplantation compared to LGE−  PHT. The presence of LV LGE was associated with lower LV EF and higher LVESVI.

This is the largest study reporting CMR findings of LGE in PHTR to date. The clinical associations described have not been previously reported. The prevalence of LV LGE in this cohort of PHTR is similar to the prevalences reported in five large adult heart transplant studies, ranging from 18 to 54% [8–11]. In four studies of PHTR undergoing CMR at the time of scheduled, surveillance EMB, LV LGE was noted to be less common (present in 0–8% of cases) [12–15]. A recent study by Soslow et al., in which CMR was performed with indication of either surveillance or clinical concern for rejection, a higher LGE prevalence was reported: 67% in PHTR with acute rejection and 56% in PHTR without acute rejection [16]. We suspect that patient selection factors—such as the inclusion or exclusion of patients undergoing a CMR for a clinical concern rather than for scheduled surveillance—explain much of the variation in LGE prevalences reported to date. In our study, which included mixed study indications, the presence of a clinical concern was associated with a higher rate of LGE positivity. We hypothesized that LGE would potentially be associated with prior comorbidities such as history of CAV and with history of moderate to severe rejection. Although these comorbidities occurred with greater frequency in the LGE + group, compared to the LGE- group, these differences did not reach statistical significance. We suspect that in a larger cohort the differences in these patient comorbities would be statistically significant.

We noted that LGE + PHTR were older than LGE- PHTR, with greater time since transplantation. In addition, age was associated with risk of LGE, independent of time since transplantation. The increased prevalence of LGE among older PHTR coincides with the known peak in risk of graft failure in PHT during late adolesence and early adulthood [23]. Notably, we did not detect a difference in donor age at time of transplant between LGE + and LGE−  patients, which suggests that the age-related risk of LGE is most likely conferred in the post-transplant course rather than from having received an older graft.

Presence of LGE was associated with lower LV EF and higher LVESVI. Adult heart transplant recipients with LGE show similar differences in LV functional parameters as well as increased rates of subsequent adverse events including major adverse cardiac events (MACE) and death [8–11]. The observed differences in LV EF and LVESVI in patients with LGE in this pediatric cohort adds to the evidence that LGE is not a benign finding. The presence of LGE in PHTR is likely multifactorial, resulting from more than one potential, prior disease process. The resultant myocardial changes and adverse outcomes found in adult cohorts suggests that PHT with LGE may be at increased risk of clinical deterioration. The addition of CMR to the routine surveillance of PHTR confers the benefits of tissue characterization throughout the myocardium, and is likely to improve risk stratification as more long-term outcomes data become available.

Scrutiny of observed patterns of LGE may provide clues about the etiology of these myocardial injuries. LGE patterns in adult heart transplant recipients have traditionally been classified as infarct-typical or infarct-atypical. To date, the best evidence tying an LGE pattern to a specific disease process affecting heart transplant recipients involves CAV. In multiple adult studies, the infarct-typical, subendocardial pattern of LGE was associated with CAV, while infarct-atypical LGE was not [10, 18, 24]. In our population with an overall low CAV prevalence (11%), which was primarily low-grade CAV, we observed LGE that was nearly exclusively infarct-atypical (97% of LV LGE + cases within the entire cohort). Even among PHTR with CAV, infarct-atypical LGE predominated. We found only one case of infarct-typical LGE, in a follow-up study of a patient with interval development CAV 3; LGE was found in the expected distribution of the stenotic coronary artery in that patient. Because infarct-typical LGE patterns are more likely to occur with increasing CAV grade [10, 24], the predominance of low-grade CAV among CAV + patients in this cohort may explain why we observed so little infarct-typical LGE.

The LV LGE patterns we observed could be classified into three distinct, non-mutually exclusive categories. Inferoseptal (posterior RV insertion point) enhancement was the most common, followed by lateral LV wall. Diffuse enhancement (involving four or more AHA segments) was the least common. Inferoseptal enhancement was frequently found in isolation, without extensive LGE involvement elsewhere in the LV myocardium. Lateral wall enhancement was generally subepicardial and infarct-atypical. Notably, the inferoseptal enhancement observed was remote from the RV-aspect of the mid-septal region typically accessed for endomyocardial biopsy, involvement of other septal segments was rare (Fig. 1). As such, the observed LGE patterns are not consistent with iatrogenic scarring secondary to prior biopsy. While we suspect that different LGE patterns are associated with different disease processes or transplant comorbidities, this study was inadequately powered to test for those differences. Further research into these findings would aid in understanding the clinical significance of the observed LGE patterns.

For patients with LV LGE by qualitative assessment, the average LGE burden by subsequent quantiative analysis was 18% of LV mass. This LV LGE burden is marginally higher than the means (3–12.2%) previously reported among adult heart transplant recipients [8–11]. Soslow et al. reported 12.3% and 13.7% LGE mass in PHTR with and without acute rejection, respectively, in the only prior report of LGE quantification among PHTR [16]. However, our quantification data were not highly reproducible. We performed repeat LV quantification in a subset of patients with LV LGE. Bland-Altman analysis revealed no significant bias between quantification iterations, but the 95% limits of agreement between the paired measurements were unacceptably high. Because of these concerns, we did not use LGE mass percentage as an outcome variable when testing for associations with patient demographics and clinical history.

We identified three primary difficulties with LGE quantification: First, the LGE mass calculation is very sensitive to small adjustments to the manual regions of interest selected for healthy and hyperenhanced myocardium in children. This is a well-described source of variability in quantification even in adults [25]. Second, precise contouring of the lateral LV epicardial border was difficult in cases with subepicardial enhancement, which was a commonly observed pattern in this cohort. Third, there is no consensus regarding the best LGE thresholding technique (such as full-width half-maximum, n-SD, or peak remote myocardium). These methods are known to produce different results [26]. We propose that quantification methods must become more robust and standardized in order to improve their clinical and research utility.

Future inquiry should include longitudinal studies of PHTR undergoing serial CMR studies, with correlation to interval events. Any identified associations between graft disease processes and specific LGE patterns would aid in the early and non-invasive identification of patients requiring increased monitoring or treatment. Specifically, future study should include consideration of a multi-center study of PHTR undergoing CMR.

## Limitations

This study’s retrospective, single-center design and sample size limits generalizability to other PHTR cohorts. Specifically, there is selection-bias in the PHTR who were referred for CMR, which was prompted by a clinical concern in some cases. The sample size limits this study’s power to assess differences in clinical history between LGE + and LGE- patients. Longitudinal clinical outcomes were not assessed. Quantification of LV LGE mass percentage was not highly reproducible for the reasons discussed above. Because only one patient had RV LGE on initial CMR, this study was underpowered to assess differences in RV volume index and RV EF based specifically on the presence of RV LGE.

## Conclusion

CMR-derived LGE was noted in a third of PHTR. Older PHTR and those with greater time since transplantation were more likely to have LGE, with age being an important predictor of LGE independent of time since transplant. Grafts with LGE had lower mean LV EF. LGE may be an important finding in surveillance for graft failure and further investigation is needed to determine its association with clinical outcomes.

## Data Availability

The datasets used and/or analyzed during the current study are available from the corresponding author on reasonable request

## Abbreviations

ACR: Acute cellular rejection
AHA: American Heart Association
AMR: Antibody mediated rejection
CAV: Cardiac allograft vasculopathy
CMR: Cardiovascular magnetic resonance
HLA-PRA: Human leukocyte antigen—panel reactive antibody
IRB: Institutional review board
HIPAA: Health Insurance Portability and Accountability Act
LGE: Late-gadolinium enhancement
LV: Left ventricle/left ventricular
LVEDVI: Left ventricular end-diastolic volume index
LVESVI: Left ventricular end-systolic volume index
LVMI: Left ventricular mass index
LV EF: Left ventricular ejection fraction
PCWP: Pulmonary capillary wedge pressure
PHTR: Pediatric heart transplant recipient(s)
RV: Right ventricle/right ventricular
RVEDVI: Right ventricular end-diastolic volume index
RVESVI: Right ventricular end-systolic volume index
RV EF: Right ventricular ejection fraction
SD: Standard deviation
